# Effects of Dietary Selenium Yeast Supplementation on Lactation Performance, Antioxidant Status, and Immune Responses in Lactating Donkeys

**DOI:** 10.3390/antiox13030275

**Published:** 2024-02-24

**Authors:** Manman Tong, Shuyi Li, Fang Hui, Fanzhu Meng, Li Li, Binlin Shi, Yanli Zhao, Xiaoyu Guo, Yongmei Guo, Sumei Yan

**Affiliations:** Inner Mongolia Key Laboratory of Animal Nutrition and Feed Science, College of Animal Science, Inner Mongolia Agricultural University, Hohhot 010018, China; tongmm@emails.imau.edu.cn (M.T.); lsy2835245597@emails.imau.edu.cn (S.L.); hf2021@emails.imau.edu.cn (F.H.); fanzhumeng@emails.imau.edu.cn (F.M.); lily972021@emails.imau.edu.cn (L.L.); shibl@imau.edu.cn (B.S.); ylzhao2017@imau.edu.cn (Y.Z.); gxy2024@imau.edu.cn (X.G.); ymguo2020@imau.edu.cn (Y.G.)

**Keywords:** selenium yeast, lactating donkeys, lactation performance, antioxidant status, immune responses

## Abstract

Donkey milk is a traditional medicinal food with various biological activities. However, its production is very low, and lactating donkeys often experience oxidative stress, leading to a further decline in milk yield. In this study, we supplemented the diets of lactating donkeys with yeast selenium (SY) to investigate its effects on lactation performance, antioxidant status, and immune responses, and we expected to determine the optimum additive level of SY in the diet. For this study, 28 healthy lactating Dezhou donkeys with days in milk (DIM, 39.93 ± 7.02 d), estimated milk yield (EMY, 3.60 ± 0.84 kg/d), and parity (2.82 ± 0.48) were selected and randomly divided into 4 groups of 7 donkeys in each: Group SY-0 (control), Group SY-0.15, Group SY-0.3, and Group SY-0.5, with selenium supplementation of 0, 0.15, 0.3, and 0.5 mg of Se/kg DM (in form of SY) to the basal diet, respectively. The results showed a dose-dependent increase in milk yield, milk component yield, milk protein production efficiency, milk production efficiency, the activities of glutathione peroxidases (GSH-Px), catalase (CAT), and total antioxidant capacity (T-AOC), as well as the content of serum interleukin-10 (IL-10), white blood cells (WBC), lymphocytes (LYM), red blood cells (RBC), hematocrit, plasma selenium, and milk selenium. Conversely, it presented a dose-dependent decrease in the activity of nitric oxide synthase (iNOS) and the contents of malondialdehyde (MDA), reactive oxygen species (ROS), nitric oxide (NO), interleukin-1β (IL-1β), interleukin-6 (IL-6), and interferon-γ (IFN-γ). In conclusion, the results confirmed that dietary supplementation with SY can improve lactation performance, antioxidant status, and immune responses in lactating donkeys, and the recommended dose of SY was 0.3 mg/kg.

## 1. Introduction

Donkey milk has a wide and long tradition of use around the world. In Chinese minority medicine, there are records of donkey milk being used to treat tuberculosis, liver cirrhosis, gastric ulcer, whooping cough, and other diseases [1]. In the Greek tradition, donkey milk is commonly fed to infants when breast milk is insufficient and is also used to treat infantile cough [2]. In Italy, donkey milk is believed to have youth-preserving and disease-curing properties [3]. Recent studies suggest that donkey milk has a relatively high nutritional value. Compared to ruminant milk, donkey milk has a higher whey protein percentage and is richer in lysozyme, lactoferrin, vitamin C, and vitamin D, meanwhile its composition is more similar to that of human milk [4,5]. In addition, many studies have shown that donkey milk has biological activities, such as antibacterial [6], antitumor [7], and anticancer [8], antioxidant [9], immunomodulatory [10] and hypoallergenic [11], showing good potential in the maintenance of health and treatment of human diseases.

In recent years, donkey milk has received increasing attention from researchers and consumers. However, restricted to the extremely underdeveloped milk cistern of the mammary glands, the donkey’s milk yield remains low. The milk yield of donkeys was reported as about 1–1.5 kg/d [12], which was much lower compared to dairy cows (generally more than 25 kg/d). Methods of improving the lactation performance of donkeys have become a focus topic in recent years. The dairy performance of livestock, including milk yield and milk quality, is influenced by various factors, such as physiological structure, genetic basis, lactation stage, nutrition, and feeding environment. During lactation, the rapid development of the mammary glands and the synthesis of large quantities of milk constituents lead to a significant increase in energy requirement and metabolic level, resulting in an increased risk of oxidative stress, which will limit the lactation potential [13,14]. Proper nutritional regulation during lactation may be an effective method to relieve oxidative stress and improve the health and lactation performance of lactating donkeys.

Selenium (Se) is an essential trace element for humans and animals. It is involved in many physiological processes in the body, and Se is the structural component of at least 25 currently known selenoproteins, such as glutathione peroxidases (GSH-Px), thioredoxin reductases, iodothyronine deiodinases, selenoprotein K, and selenoprotein P, which play important physiological functions in maintaining cellular antioxidant status, alleviating inflammation, and regulating immunity. In livestock, Se deficiency causes a wide range of disorders in growth, reproduction, and health [15]. Alhidary et al. [16] found that limiting Se supply (e.g., lower feed intake, Se deficiency in feed) or increasing Se depletion (e.g., peak lactation) would limit antioxidant capacity. It has also been reported that dietary supplementation with Se can improve the lactation performance [17], antioxidant status [18], and immune function [17] of dairy cows. Chen et al. [19] showed that increasing Se supply could improve milk composition and antioxidant status in sows and promote the transfer of immunoglobulins from blood to milk. These studies suggest that appropriate doses of dietary Se may be an effective method of regulating health and lactation performance in donkeys. However, little research is available on the effects of dietary Se levels on lactating donkeys, so it is still unclear whether the addition of Se has positive impacts on lactation performance and antioxidant and immune status.

Studies have shown that selenium is a potent antioxidant [20,21]. Selenium yeast (SY), selenate or seleniteis are common dietary sources of Se supplementation. Selenomethionine (SeMet) is the main form of Se in SY. Dietary Se from SY is more easily incorporated in animal tissues than inorganic Se [22]. Previous studies have shown that SY can effectively increase Se content in milk and blood [23] and improve the antioxidant capacity [14] in dairy cows. However, the effects of adding SY to the diets of lactating donkeys have not been previously reported. The hypothesis of this study is that appropriate supplementation of dietary SY is benefit to lactation performance, antioxidant and immune levels in lactating donkeys. Therefore, the aim of our current study was to investigate the effects of dietary SY supplementation on lactation performance and antioxidant and immune parameters and to expect to obtain the optimum additive level of SY in the diets of lactating donkeys.

## 2. Materials and Methods

### 2.1. Ethics Statement

This study was conducted at Inner Mongolia Grassland Yulv Science and Technology Animal Husbandry Co., Ltd. (Hohhot, China). All procedures were approved by the Animal Ethics and Welfare Committee at the Inner Mongolia Agricultural University (NND2021050) and were under the university’s guidelines for animal research.

### 2.2. Experimental Design and Treatments

A total of 28 healthy lactating Dezhou donkeys were selected according to estimated milk yield (EMY, 3.60 ± 0.84 kg/d), days in milk (DIM, 39.93 ± 7.02 d), age (6.01 ± 0.54 year), parity (2.82 ± 0.48), and body weight (247.24 ± 26.27 kg) and randomly divided into 4 groups of 7 donkeys in each: Group SY-0 (control), Group SY-0.15, Group SY-0.3, and Group SY-0.5 with Se supplementations of 0, 0.15, 0.3, and 0.5 mg of Se/kg DM to the basal diet, respectively. The basal diet contains 0.04 mg of Se/kg DM without extra Se supplementation. In this trial, Se was supplemented in the form of SY (SelenoSource AFTM2000, Diamond V Biological Fermentation Engineering & Technologies Shenzhen Co., Ltd.; Shenzhen, China), which contained 2000 mg/kg of Se. SeMet is the main form of Se in SY. Ingredients of the diet and nutrient content of the basal diet are presented in Table 1. The forage-to-concentrate ratio of the diets offered to donkeys was 70:30. All lactating donkeys were housed in individual pens keeping visual, vocal, and olfactory contact with each other. The experiment included a 2-week pretrial period for adaptation and an 8-week experimental period for data and sample collection.

#### 2.2.1. Feeding Management

The donkeys were fed concentrate, corn silage and alfalfa at 7:00 a.m. and 2:00 p.m. every day. Millet straw was fed five times a day. Feed refusals were removed, weighed, and recorded daily before the morning feeding. The amount of feed offers was adjusted to ensure a 5% to 10% refusal. Donkeys were housed with the foals, but from 7:00–10:00 a.m. (3 h) and 2:00–5:00 p.m. (3 h) daily, the foals were separated from the mares while maintaining visual, vocal and olfactory contact. Donkeys were milked twice daily at 10:00 a.m. and 5:00 p.m., and the milking yield was recorded for every donkey. All the donkeys were allowed ad libitum access to water.

#### 2.2.2. Sampling Procedures

##### Feed Sampling

During the experiment, the feed offers and refusals were recorded daily for each donkey for individual dry matter intake (DMI) calculations and collected as the feed samples for the nutritional and total Se concentration analysis. All feed samples were pooled and dried at 65 °C for 72 h until constant weight was achieved and then ground by a pulverizer (Arthur H. Thomas Co., Philadelphia, PA, USA) to be passed through a 1 mm screen for subsequent analysis.

##### Milk Sampling

Milk samples were collected weekly and samples from the morning and afternoon milkings (10:00 a.m. and 17:00 p.m.) of every donkey were mixed in a 1:1 ratio. The mixed samples were used for the subsequent determination of milk composition and somatic cell count (SCC) of each donkey. In addition, one part of mixed milk from the 8th week was frozen at −20 °C for Se concentration analysis.

##### Blood Sampling

Blood samples were collected at week 8 prior to morning feeding (7:00 a.m.) via the jugular vein with blood collection tubes (Corning Incorporated Costar, Corning, NY, USA). The blood samples in EDTA collection tubes were taken for hematological parameter detection. Blood samples in ordinary collection tubes and heparin sodium collection tubes were centrifuged at 3000× *g* at 4 °C for 15 min to separate the serum and plasma, respectively, which were stored at −20 °C until analysis.

#### 2.2.3. Sample Analysis

##### Feed Analysis

Feed samples were analyzed for DM (method 930.15), crude protein (CP, method 984.13) and ether extract (EE, method 920.39), and calcium and phosphorus (method 935.13) according to the methods of the Association of Official Analytical Chemists (AOAC International, 2000). Neutral detergent fiber (NDF) and acid detergent fiber (ADF) were determined according to the methods of Van Soest et al. (1991) [24] with an Ankom 220 Fiber Analyser (Ankom Co., Macedon, NY, USA), and sodium sulfite was used and expressed exclusive residual ash.

##### Lactation Performance and Somatic Cell Count

The contents of milk protein, fat, lactose, urea-nitrogen (MUN), total solids (TS), and solid-not-fat (SNF) were determined using infrared spectrophotometry (Milkoscan FT+, Foss Analytical Co., Ltd., Hillerød, Denmark) and SCC was analyzed by an automatic somatic cell counter (Foss-somatic FC, Foss Analytical Co., Ltd., Hillerød, Denmark). Milk yield was calculated as follows:

- Estimated milk yield (EMY, kg/d) = milking yield (the sum of the milking yield twice a day) (kg/d) × 4 [25].
- Solids-corrected milk (SCM, kg/d) = {(12.3 × milk fat (%) content of non-standard milk + 6.56 × solid-not-fat (%) content of non-standard milk − 0.0752)} × EMY (kg/d) [26].
- Energy-corrected milk (ECM, kg/d) = 0.327 × EMY (kg/d) + 12.95 × fat (kg/d) + 7.65 × protein (kg/d) [27].
- Milk composition yield = milk composition (%) × milking yield (kg/d).
- Feed conversion rate was expressed as follows: EMY (kg/d)/DMI (kg/d), SCM (kg/d)/DMI (kg/d) and ECM (kg/d)/DMI (kg/d) [26].
- Milk protein synthesis efficiency = {EMY (kg/d) × milk protein (%)}/{DMI (kg/d) × CP level (%)} [28].

##### Hematological Parameters

An automated blood cell analyzer (ADVIA 2120, Siemens Healthcare Diagnostic, Munich, Germany) was used to analyze the following hematological parameters: the count of white blood cells (WBC), neutrophils (NEU), lymphocytes (LYM), intermediate cells (MID), red blood cells (RBC), and platelets (PLT); hemoglobin concentration (HGB); hematocrit (HCT); and neutrophil-to-lymphocyte ratio (NLR).

##### Antioxidant Indicators

Serum samples were analyzed for antioxidant indicators, including GSH-Px activity, superoxide dismutase (SOD) activity, catalase (CAT) activity, total antioxidant capacity (T-AOC), and malondialdehyde (MDA) content using commercial detection kits (A005-1-2, A001-1-2, A007-1-1, A015-1-2, A003-1-2; Nanjing Jiancheng Bioengineering Institute, Nanjing, China) following the manufacturer’s instructions. The concentration of reactive oxygen species (ROS) was detected at the Beijing SINO-UK Institute of Biological Technology using commercial assay kits (Beijing SINO-UK Institute of Biological Technology, Beijing, China).

##### Immune Indicators

The concentrations of interleukin (IL)-1β, IL-2, IL-4, IL-6, IL-10, tumor necrosis factor-α (TNF-α), and interferon-γ (IFN-γ) were detected at the Beijing SINO-UK Institute of Biological Technology, using enzyme-linked immunosorbent assay kits (Beijing SINO-UK Institute of Biological Technology, Beijing, China) on a microplate reader (DR-200BS, Hiwell-Diatek Instruments (wuxi) Co., Ltd., Wuxi, China). Nitric oxide (NO) and nitric oxide synthase (iNOS) were determined using commercial detection kits (A012-1-2, A014-1-2; Nanjing Jiancheng Bioengineering Institute, Nanjing, China).

##### Selenium Content Analysis

Mixed feed samples, every donkey’s milk, and plasma samples were used for Se concentration analysis according to the methods of the National Standard of the People’s Republic of China: National Food Safety Standard-Determination of Multi-elements in Foods (Stands Press of China, Beijing; GB5009.268-2016 [29]) with modifications. Briefly, 2 g of feed samples (accurate to 0.001 g), 0.8 g of freeze-dried milk, or 1 mL of plasma was accurately measured, and then 20 to 30 mL of mixed acid (nitric acid:perchloric acid = 2:1) was added for digestion, left for 1 h or overnight to incubate, and then heated on an electric heating plate at 300 °C until the liquid was clear and colorless or slightly yellow. The mixed solution was diluted to an appropriate volume and then used to analyze Se concentration utilizing inductively coupled plasma optical emission spectrometry (ICP-OES) (ICAP 6300Duo, Thermo Fisher Scientific, Waltham, MA, USA). The ratio of milk to plasma Se is calculated thusly: milk Se content (ug/L)/plasma Se content (ug/L) × 100.

#### 2.2.4. Statistical Analysis

All statistical analyses were performed in SAS software (version 8.0, SAS Institute Inc., Cary, NC, USA). The indicators of lactation performance and SCC were analyzed with the PROC MIXED procedure as follows:

Yijkm = μ+ Ci + Wj+ Ci Wj + bXjk + Sim + εijkm,

where Yijkm = the dependent variable; μ = overall mean; Ci = fixed effect of dietary Se levels, Wj = fixed effect of lactation week (j = 1 to 8 at weeks 1, 2, 3, 4, 5, 6, 7, and 8), Ci Wj = effect of the interaction between diet treatment and lactation week, bXjk = effect of covariate (week 0, the observations during the 2 weeks of pretrial period served as covariates for the corresponding experimental period), Sim = a random effect (individual donkey); εijkm = residual error.

Hematological parameters, antioxidant indicators, immune indicators, plasma Se content, milk Se content, and the ratio of milk to plasma Se were analyzed using the GLM procedure on normally distributed data, otherwise using Kruskal–Wallis test. Differences between treatments were evaluated by Duncan’s multiple range test. Meanwhile, multivariate regression analysis was conducted to evaluate the linear and quadratic effects of the increasing levels of SY on the various indices. Data are expressed as least squares means and standard errors of the means. Probability values of *p* ≤ 0.05 were used to define statistical significance.

## 3. Results

### 3.1. Lactation performance

The effects of different levels of SY in the diet on DMI, lactation performance, and SCC of lactating donkeys are shown in Table 2. Compared to group SY-0, estimated milk yield (EMY) was increased significantly (*p* < 0.05) in the SY-0.3 and SY-0.5 groups, the SY-0.3 group was better than the SY-0.15 group (*p* < 0.05), and there were no significant differences between the other groups (*p* > 0.05). The variations of SCM, ECM, protein yield, lactose yield, SNF yield, and TS yield with the addition of SY to the diet were the same as EMY. Fat yield was improved (*p* = 0.025) in the SY-0.3 and SY-0.5 groups compared to the SY-0 group, while no significant differences were found between the SY-0.15 group and all other groups (*p* > 0.05), and the SCC in the milk showed the opposite results (*p* = 0.018). EMY/DMI, SCM/DMI, and ECM/DMI in the SY-0.3 and SY-0.5 groups were significantly higher than those in the SY-0 and SY-0.15 groups (*p* < 0.05), with non-significant differences between groups SY-0.3 and SY-0.5 (*p* > 0.05) or groups SY-0 and SY-0.15 (*p* > 0.05). Donkeys in the SY-0.3 group had a better milk protein synthesis efficiency than the other three groups (*p* = 0.001).

With the increase of SY supplementation, EMY, SCM, ECM, lactose yield, SNF yield, TS yield, and milk protein production efficiency showed a linear (*p* < 0.05) and a quadratic (*p* < 0.05) increase and reached the highest value in SY-0.3; fat yield, protein yield, EMY/DMI, SCM/DMI, and ECM/DMI showed a significant linear increase (*p* < 0.05), and SCC showed a significant linear decrease (*p* = 0.003). DMI and the contents of fat, protein, lactose, SNF, TS, and MUN were not affected by the dose of SY (*p* > 0.05).

### 3.2. Hematological Parameters

As shown in Table 3, the count of WBC and LYM and the percentage of LYM in the blood of the donkeys increased quadratically (*p* < 0.05) with increasing supplementation of dietary SY and reached the highest value in the SY-0.3 group compared to the other groups (*p* < 0.05), while no significant changes were observed in the SY-0, SY-0.15, and SY-0.5 groups (*p* > 0.05). With increased SY supplementation in the diet of donkeys, the RBC and HCT in the blood were improved linearly (*p* = 0.013, *p* = 0.002) and quadratically (*p* = 0.003, *p* = 0.000) significantly. The blood RBC of donkeys reached the highest value in the SY-0.3 and SY-0.5 groups, which is significantly higher than the SY-0 group, and no significant changes were found between the SY-0.15 group and all other groups (*p* > 0.05). Similarly, the blood HCT was raised remarkably in groups SY-0.15, SY-0.3, and SY-0.5 compared to the SY-0 group (*p* = 0.001), and the group SY-0.3 was better than SY-0.15 (*p* = 0.001). Different groups had no changes in the other hematological parameters (*p* > 0.05).

### 3.3. Antioxidant Indicators in Serum

As presented in Table 4, the activities of GSH-Px, CAT, and T-AOC in the serum increased linearly (*p* < 0.05) and quadratically (*p* < 0.05) with the supplementation of SY to the diet of lactating donkey. Adding SY improved serum GSH-Px activity in the SY-0.15, SY-0.3, and SY-0.5 groups compared with the SY-0 group (*p* = 0.000), and no statistically significant changes were found among the above three groups (*p* > 0.05). The activity of CAT in the serum was significantly higher in the SY-0.3 and SY-0.5 groups than in the SY-0 and SY-0.15 groups (*p* = 0.000), with non-significant changes between groups SY-0.3 and SY-0.5 (*p* > 0.05), groups SY-0 and SY-0.15 (*p* > 0.05). Compared to the SY-0 group, T-AOC in the serum was enhanced (*p* = 0.018) in the SY-0.3 and SY-0.5 groups, and there were no significant differences between the other groups (*p* > 0.05).

Additionally, the contents of MDA and ROS significantly decreased linearly (*p* = 0.014, *p* = 0.015) and quadratically (*p* = 0.009, *p* = 0.025) with the supplementation of SY to the diet, and it reached the lowest value in the SY-0.3 and SY-0.5 groups. Compared to groups SY-0 and SY-0.15, the contents of MDA and ROS in groups SY-0.3 and SY-0.5 significantly decreased (*p* = 0.023, *p* = 0.032), and there were no significant differences between groups SY-0.3 and SY-0.5 (*p* > 0.05) or groups SY-0 and SY-0.15 (*p* > 0.05). There were no significant differences in serum SOD activity among the groups (*p* > 0.05).

### 3.4. Immune Indicators in Serum

As presented in Table 5, the contents of NO, IL-1β, IL-6, and IFN-γ and the activity of iNOS in serum showed a linear (*p* < 0.05) and a quadratic (*p* < 0.05) decrease with increasing supplementation of SY to the lactating donkey diet. The diets of the groups SY-0.15, SY-0.3, and SY-0.5 significantly reduced serum iNOS activity compared to the SY-0 group, and the value in the SY-0.3 group is the lowest, which is remarkably lower than that in the SY-0.15 and SY-0.5 groups (*p* = 0.000). Serum NO content significantly decreased in the SY-0.3 and SY-0.5 groups compared to the SY-0 group (*p* = 0.023), and no differences were found between the other groups (*p* > 0.05). Serum IL-6 showed a similar trend to NO (*p* = 0.005). Additionally, serum IL-1β content significantly decreased in the SY-0.3 and SY-0.5 groups as compared with the groups SY-0 and SY-0.15 (*p* = 0.001), however, the differences between the SY-0.3 and SY-0.5 groups (*p* > 0.05) and the SY-0 and SY-0.15 groups (*p* > 0.05) were not significant. Serum IFN-γ content (*p* = 0.000) had similar results to IL-1β.

With the addition of SY to the diet of lactating donkeys, serum IL-10 content showed a linear (*p* = 0.048) and a quadratic (*p* = 0.000) increase, and the SY-0.3 group had the highest values compared to the other groups, and the SY-0.5 group was lower than the SY-0.3 group but higher than the SY-0 and SY-0.15 groups (*p* < 0.001). The supplementation of dietary SY had no significant effect on other serum immune indicators (IL-2, IL-4, and TNF-α) (*p* > 0.05).

### 3.5. Selenium Concentrations in Milk and Plasma

As presented in Table 6, with increasing SY supplementation to the diet, the concentrations of plasma Se and milk Se and the ratio of milk to plasma Se in week 8 showed a significant linear (*p* < 0.05) and quadratic (*p* < 0.05) increase. The concentration of plasma Se had the highest value in the SY-0.5 group and the lowest value in the SY-0 group, and the value in the SY-0.3 group is significantly bigger than in the SY-0.15 group (*p* < 0.001). The milk Se concentration changed in the same pattern. The ratio of milk to plasma Se in the SY-0.3 and SY-0.5 groups was highest—significantly higher than the SY-0 and SY-0.15 groups (*p* < 0.001)—while the SY-0.15 group was higher than the SY-0 group (*p* < 0.001), and there were no changes between groups SY-0.3 and SY-0.5 (*p* > 0.05). There were no differences between groups in the concentrations of plasma Se and milk Se and the ratio of milk-to-plasma Se at week 0.

## 4. Discussion

Previous studies on the effect of Se supplementation on lactation performance have mainly focused on dairy cows [17], sheep [30], and sows [18]. However, there have been no reports regarding the genus *Equus*. Our study was the first investigation into the effects of SY on lactating donkeys. The results of the present study indicated that with increasing dietary SY supplementation in lactating donkeys, milk yield, milk component yield, milk production efficiency, and milk protein synthesis efficiency showed a dose-dependent increase, and the additive levels of 0.3 and 0.5 mg/kg were found to be preferable, with 0.3 mg/kg being the favored option. The study conducted by Hachemi et al. [31] showed that supplementing the diet with SY increased milk production in mid-lactation dairy cows. Li et al. [17] found that the addition of hydroxyselenomethionine to the diet increased milk yield, milk protein and lactose production, and milk production efficiency in early-lactation dairy cows. Their findings were basically consistent with the results of our study. In addition, the increase in lactose production is a sign that lactation is activated. Lactose plays a role in regulating the osmotic pressure of secretory vesicles in mammary epithelial cells, so the amount and efficiency of lactose synthesis determine milk production [32]. In this experiment, both the lactose production and milk yield of donkeys were significantly increased, suggesting that these data were well supported by each other.

Lactating animals often experience some degree of oxidative stress [13,14]. Improving their antioxidant status is a possible way to enhance lactation performance [33]. The GSH-Px is an important Se-dependent antioxidant enzyme in mammals [34]. In addition, CAT and T-AOC are also indicators used to evaluate antioxidant capacity [35,36]. The contents of free radicals (e.g., ROS) and lipid peroxides (e.g., MDA) reflect the level of oxidative stress, and their over-accumulation can cause further damage to cells and tissues [36]. In the present study, adding 0.3 to 0.5 mg/kg of SY to the diet of lactating donkeys increased the activities of GSH-Px, CAT, and T-AOC and decreased the contents of MDA and ROS in serum significantly, and there existed a dose-dependent relationship between the indices and SY doses. Studies on different animals (such as dairy cows and dairy goats) showed that dietary Se supplementation could significantly promote antioxidant capacity and alleviate oxidative stress in livestock [33]. The available reports on the effect of Se on the antioxidant function of *Equus* are severely limited. Brummer et al. [37] assessed the effect of Se concentration changes on the antioxidant status of adult horses, and the addition of 0.3 mg/kg of Se increased blood Se concentrations and blood GSH-Px activity in adult horses. White et al. [38] discovered that 0.3 mg/kg Se supplementation in adult horse diets might help mitigate oxidative muscle damage after prolonged exercise and contribute to post-exercise recovery. Their studies support our results. Furthermore, Wang et al. [39] found that there was a positive relationship between serum Se concentration and GSH-Px activity [39]. Similar results were found in this study that both plasma Se concentration and GSH-Px activity increased with increasing dietary supplementation of SY. Dietary Se supplementation can reduce the oxidation of unsaturated fatty acids in whole blood [40], which may protect the membrane of RBC from lipid peroxidation and promote the production of RBC [41]. Therefore, the results of increased RBC and HCT in the blood in our study also indicated an improvement in antioxidant status. In summary, dietary SY supplementation enhanced the antioxidant function of lactating donkeys. Selenomethionine (SeMet) is the main form of Se in SY. SeMet has an extremely strong redox activity under physiological conditions [42] and can bind to proteins and enzymes instead of methionine, thus conferring additional redox activity to these proteins [43]. Therefore, this may also be one of the reasons that SY can increase antioxidant level.

Oxidative stress and inflammation response are closely related, often triggering and promoting each other [44]. NO is a reactive nitrogen mainly catalyzed and produced by iNOS [45]. When the body is under inflammation, large amounts of NO are released. The massive release of NO leads to the production of pro-inflammatory cytokines such as IL-1β, IL-2, IL-6, TNF-α, and IFN-γ [46]. IL-4 and IL-10 are common anti-inflammatory cytokines that are upregulated in response to inflammation to alleviate inflammation [46]. In our study, the findings indicated that supplementing SY of 0.3 and 0.5 mg/kg resulted in a dose-dependent decrease in the activity of iNOS and the contents of NO, IL-1β, IL-6, and IFN-α in the serum and a dose-dependent increase in the content of IL-10 in the serum, suggesting that the addition of SY exerted a certain anti-inflammatory effect. Some studies have shown that Se deficiency and the inhibition of selenoprotein expression are associated with elevated concentration of pro-inflammatory cytokines in various tissues, including the gastrointestinal tract, uterus, breast tissue, and other tissues [35]. Studies have shown that Se supplementation inhibits inflammatory changes caused by *S. aureus*, including down-regulation of IL-1β, IL-6, and TNF-α concentration [47]. The above findings support the results of our study. Hence, the addition of SY did improve the immunomodulatory capacity of lactating donkeys in this trial. Enhancing immune function can reduce oxidative stress and the risk of inflammation, thereby promoting udder health and contributing to lactation capacity [33].

The health of the mammary gland has a direct impact on lactation performance. The SCC is a basic indicator used to assess the health of the mammary glands and lactation performance [33]. In the present study, the supplementation of SY to the diet in lactating donkeys resulted in a linear decrease in SCC, and doses of 0.3 and 0.5 mg/kg were found to be more effective. Se supplementation plays an important role in reducing oxidative stress, protecting udder health, and decreasing the incidence of mastitis [33,48]. Various studies on dairy cows [31,39] and dairy goats [48,49] have shown that Se supplementation can reduce SCC in milk, which is consistent with our results. The reduction in SCC indicated that the addition of SY in this trial improved mammary health in lactating donkeys, which probably resulted from improved antioxidant and immune function. In addition, the main component of SY, SeMet, has been shown to reduce apoptosis in bovine mammary epithelial cells [50]. In addition, certain selenoproteins have the ability to reduce the gene expression of some pro-inflammatory cytokines, thereby reducing the inflammatory response [51]. These factors may explain the positive effects of SY supplementation on SCC.

Furthermore, in the present study, we detected hematological parameters and observed a dose-dependent increase in the counts of WBC, LYM, RBC, and HCT and the percentage of LYM with increasing dietary SY supplementation in lactating donkeys. Additionally, the optimal level of supplementation was found to be 0.3 mg/kg. The counts of WBC, LYM, and RBC in the blood can reflect the immune function of the cows, and a proper increase in these counts indicates an enhancement of immunity [52,53]. Li et al. [17] found that 0.1 and 0.3 mg/kg of hydroxyselenomethionine significantly increased the counts of WBC and RBC in early-lactation dairy cows. The research of Hachemi et al. [31] showed that 0.3 mg/kg SY increased RBC significantly in mid-lactation dairy cows. These reports supported our results to some extent. The increase in WBC and RBC may be attributed to reduced oxidative stress and maintenance of cell membrane integrity, and it suggested that SY had a beneficial effect on antioxidant capability and immune status [17]. Although blood RBC and HCT increased linearly and quadratically with the increase of the dose of SY, the quadratic curves have more significant *P* values. This showed that it wasn’t the higher the dosage of additives, the better. Our results showed that the blood RBC and HCT in the SY-0.5 group did not improve further compared to the SY-0.3 group and even showed a downward trend. This is consistent with the notion that high levels of antioxidant additives may reduce their antioxidant capacity [40]. Furthermore, the linear and quadratic significant differences mainly because the number of doses set is not high enough. If we set more or higher doses, we might obtain a more precise result. All the changes in hematological parameters in this study were within the normal range [54,55]. The addition of different doses of SY in our study did no harm to the lactating donkeys.

In this trial, SY supplementation increased plasma Se efficiently. Plasma Se content is an indicator that can reflect the Se status of the body, antioxidant capacity, and immunity level in animals [34,56]. The studies conducted by Calamari et al. [57] and Richardson et al. [58] in horses got similar results to our own research. Plasma Se can be transferred to breast tissue via the body circulation [33]. The milk Se content and the ratio of milk to plasma Se increased significantly as the level of SY additive increased, suggesting plasma Se can efficiently transfer to the milk. However, the increase in transfer efficiency slowed down when the level of Se additive reached 0.3 mg/kg. The results of Sun et al. [59] and Li et al. [17] were in agreement with our findings. Differences in transfer efficiency may be related to metabolic pathways. To sum up, the addition of SY to the diet effectively increased plasma and milk Se concentration, which may contribute to improving the body’s antioxidant level and milk quality.

In the present study, most of the indicators affected by dietary SY showed more positive effects in the SY-0.3 and SY-0.5 groups. However, compared to the SY-0.3 group, the effect of the SY-0.5 group did not further improve and even decreased slightly in some indicators. One of a possible reason for this may be that antioxidant supplements, such as selenium, can also have pro-oxidant effects at high doses [40]. Regarding lactation performance, the SY-0.3 group was found to be optimal. As for antioxidant and immune function, both the SY-0.3 and SY-0.5 groups had comparable effects. This implies that the better dose is different in different aspects. In total, we suggested that 0.3 mg/kg was a more appropriate additive dose in this experiment. In addition, the optimal dose in the diet is related to the animal’s needs. When animals are experiencing some degree of oxidative stress (e.g., early-lactation, heat stress, prolonged exercise), their Se requirements will be higher, which should be noted [17,38].

Generally, the results of the present study indicated that the addition of SY to the diet improved the lactation performance of lactating donkeys, which may be attributed to the fact that SY improved its antioxidant function, relieved inflammation, and improved mammary gland health. Improved antioxidant status may reduce energy expenditure caused by oxidative stress, and thus more energy can be used for milk synthesis [33]. Additionally, it can also help reduce damage to breast cells caused by oxidative stress [33]. The enhanced immune function reduces the risk of inflammation, which can promote the health of the mammary gland and thereby contribute to the lactation capacity of the mammary cells.

Some researchers believe that the effect of added Se on selenoproteins and some signaling pathways such as mitogen-activated protein kinase and the nuclear factor-κb may be the mechanisms by which Se mitigates oxidative stress and inflammatory responses in dairy cows [60]. It gives us ideas for our subsequent studies. We can explore these ideas in the mammary cells of lactating donkeys. In addition, Se addition has been reported to increase rumen microbial structure and nutrient digestibility [33], which may also explain the positive effects of Se addition on lactation performance and provide a direction for our further research.

## 5. Conclusions

In summary, dietary SY supplementation in lactating donkeys improved lactation performance and milk protein synthesis efficiency, as well as immune and antioxidant status. Furthermore, plasma Se and milk Se concentration were also elevated in this study. The increased antioxidant function and improved immunomodulation caused by SY may be responsible for its enhanced lactation performance in lactating donkeys. In general, the recommended dose of SY was 0.3 mg/kg.

## Figures and Tables

**Table 1:** Composition and nutrient levels of basal diet (air-dry basis).

Item	Content
Feed Ingredients, % of DM	
Millet straw	33.97
Alfalfa hay	23.55
Corn silage	12.49
Corn	15.19
Soybean meal	8.55
Corn germ meal	1.8
Distillers dried grains with solubles	1.8
Bran	0.9
NaCl	0.39
CaCO_3_	0.21
CaHPO_4_	0.66
Premix ^1^	0.50
Nutrient level, % of DM (unless noted)	
Digestible energy ^2^, MJ/kg	10.84
DM	87.21
CP	12.28
EE	2.12
NDF	47.87
ADF	28.3
Ca	1.12
P	0.36
Se, mg/kg DM	0.04

^1^ Per kilogram of premix provided the following: vitamin A (VA) 1,200,000 IU, vitamin D (VD) 250,000 IU, eitamin E (VE) 3000 IU, Fe 8 g, Cu 1.6 g, Zn 12 g, Mn 12 g, I 72 mg, Co 100 mg. ^2^ Digestible energy was calculated according to the Chinese Feed Ingredients and Nutritional Value Table (30th edition).

**Table 2:** The effects of selenium yeast supplementation on DMI, lactation performance, and SCC.

Item	SY ^1^, mg/kg (DM Basis)				SEM	*p*-Value		
	0	0.15	0.30	0.50		Treatment	Linear	Quadratic
DMI, kg/d	7.97	8.03	7.67	7.88	0.42	0.153	0.284	0.390
Milk yield, kg/d								
EMY ^2^	3.17 ^c^	3.28 ^bc^	3.78 ^a^	3.54 ^ab^	0.34	<0.001	0.000	0.023
SCM ^3^	1.78 ^c^	1.83 ^bc^	2.11 ^a^	1.97 ^ab^	0.21	0.000	0.002	0.029
ECM ^4^	1.60 ^c^	1.70 ^bc^	1.85 ^a^	1.78 ^ab^	0.19	0.002	0.002	0.029
Milk composition								
Fat, %	0.42	0.41	0.41	0.40	0.04	0.735	0.331	0.793
Protein, %	1.83	1.83	1.83	1.82	0.04	0.888	0.454	0.789
Lactose, %	7.09	7.10	7.11	7.11	0.05	0.511	0.177	0.469
SNF, %	8.97	8.99	8.98	8.98	0.06	0.924	0.893	0.813
TS, %	9.39	9.39	9.39	9.40	0.07	0.908	0.630	0.743
MUN, mg/dL	33.10	33.31	32.52	33.10	3.05	0.820	0.811	0.674
Milk component yield ^5^, g/d								
Fat yield	12.77 ^b^	13.54 ^ab^	14.95 ^a^	14.80 ^a^	2.18	0.025	0.006	0.288
Protein yield	58.60 ^c^	59.82 ^bc^	67.55 ^a^	64.56 ^ab^	6.50	0.001	0.003	0.113
Lactose yield	221.47 ^c^	229.52 ^bc^	262.92 ^a^	247.05 ^ab^	25.90	<0.001	0.001	0.026
SNF yield	279.67 ^c^	291.52 ^bc^	332.01 ^a^	309.38 ^ab^	33.07	0.000	0.001	0.015
TS yield	292.31 ^c^	305.72 ^bc^	347.48 ^a^	327.66 ^ab^	34.98	0.000	0.001	0.023
Milk production efficiency								
EMY/DMI	0.40 ^b^	0.40 ^b^	0.48 ^a^	0.45 ^a^	0.05	<0.001	0.000	0.092
SCM/DMI	0.22 ^b^	0.23 ^b^	0.27 ^a^	0.25 ^a^	0.03	<0.001	0.001	0.050
ECM/DMI	0.21 ^b^	0.21 ^b^	0.24 ^a^	0.23 ^a^	0.03	0.000	0.001	0.141
Milk protein production efficiency ^6^	0.73 ^b^	0.74 ^b^	0.85 ^a^	0.78 ^b^	0.09	0.001	0.026	0.025
SCC, ×1000 cells/mL	5.74 ^a^	5.25 ^ab^	4.39 ^b^	4.45 ^b^	0.93	0.018	0.003	0.278

^a–c^ Data with different letters in the same row indicate significant differences (*p* < 0.05). ^1^ SY = selenium yeast. ^2^ EMY: estimated milk yield (kg/d) = milking yield (the sum of the milking yield twice a day) (kg/d) × 4 [25]. ^3^ SCM: solids-corrected milk (kg/d) = {(12.3 × milk fat (%) content of non-standard milk + 6.56 × solid-not-fat (%) content of non-standard milk − 0.0752)} × EMY (kg/d) [26]. ^4^ ECM: energy-corrected milk (kg/d) = 0.327 × EMY (kg/d) + 12.95 × fat (kg/d) + 7.65 × protein (kg/d) [27]. ^5^ Milk composition yield = milk composition (%) × milking yield (kg/d). ^6^ Milk protein synthesis efficiency = {EMY (kg/d) × milk protein (%)}/{DMI (kg/d) × CP level (%)} [28].

**Table 3:** The effects of selenium yeast supplementation on hematological parameters.

Item	SY, mg/kg (DM Basis)				SEM	*p*-Value		
	0	0.15	0.3	0.5		Treatment	Linear	Quadratic
WBC, ×10^9^/L	7.67 ^b^	8.08 ^b^	8.91 ^a^	7.68 ^b^	0.26	0.004	0.581	0.036
LYM, ×10^9^/L	2.64 ^b^	2.80 ^b^	3.48 ^a^	2.82 ^b^	0.17	0.007	0.257	0.027
MID, ×10^9^/L	0.83	0.82	0.78	0.80	0.03	0.346	0.300	0.470
NEU, ×10^9^/L	4.94	4.91	5.00	4.84	0.36	0.333	0.876	0.969
LYM, %	33.42 ^b^	34.30 ^b^	37.94 ^a^	33.44 ^b^	0.84	0.014	0.501	0.015
MID, %	9.90	9.66	9.43	9.74	0.47	0.916	0.771	0.786
NEU, %	57.10	57.62	56.58	58.47	0.72	0.316	0.307	0.372
NLR	1.74	1.71	1.66	1.78	0.05	0.355	0.676	0.263
RBC, ×10^12^/L	4.83 ^b^	5.23 ^ab^	5.70 ^a^	5.41 ^a^	0.16	0.006	0.013	0.003
HGB, g/L	108.67	107.67	107.57	106.29	6.10	0.994	0.782	0.963
HCT, %	27.58 ^c^	29.84 ^b^	32.40 ^a^	31.32 ^ab^	0.74	0.001	0.002	0.000
PLT, ×10^9^/L	228.00	214.57	234.17	229.60	10.76	0.613	0.620	0.856

^a–c^ Data with different letters in the same row indicate significant differences (*p* < 0.05). Abbreviations: SY = selenium yeast, WBC = white blood cell, NEU = neutrophil, LYM = lymphocyte, MID = intermediate cell, RBC = red blood cell, PLT = platelet, HGB = hemoglobin concentration, HCT = hematocrit, NLR = neutrophil-to-lymphocyte ratio.

**Table 4:** The effects of selenium yeast supplementation on antioxidant indicators in serum.

Item	SY, mg/kg (DM Basis)				SEM	*p*-Value		
	0	0.15	0.3	0.5		Treatment	Linear	Quadratic
GSH-Px, U/mL	415.87 ^b^	536.39 ^a^	592.92 ^a^	566.64 ^a^	23.73	0.000	0.001	<0.001
SOD, U/mL	76.76	76.30	75.88	77.39	1.29	0.861	0.752	0.704
CAT, U/mL	2.51 ^b^	2.83 ^b^	3.28 ^a^	3.44 ^a^	0.13	0.000	<0.001	<0.001
T-AOC, U/mL	1.74 ^b^	1.87 ^ab^	2.06 ^a^	1.95 ^a^	0.07	0.018	0.021	0.011
MDA, nmol/mL	0.91 ^a^	0.87 ^ab^	0.83 ^b^	0.85 ^b^	0.02	0.023	0.014	0.009
ROS, IU/mL	282.10 ^a^	271.04 ^ab^	240.64 ^b^	250.01 ^b^	10.18	0.032	0.015	0.025

^a,b^ Data with different letters in the same row indicate significant differences (*p* < 0.05). Abbreviations: GSH-Px = glutathione peroxidases, SY = selenium yeast, SOD = superoxide dismutase, CAT = catalase, T-AOC = total antioxidant capacity, MDA = malondialdehyde, ROS = reactive oxygen species.

**Table 5:** The effects of selenium yeast supplementation on immune indicators in serum.

Item	SY, mg/kg (DM Basis)				SEM	*p*-Value		
	0	0.15	0.3	0.5		Treatment	Linear	Quadratic
iNOS, U/mL	15.06 ^a^	14.35 ^b^	13.52 ^c^	14.19 ^b^	0.21	0.000	0.012	0.000
NO, umol/L	80.04 ^a^	72.56 ^ab^	68.28 ^b^	70.01 ^b^	2.65	0.023	0.013	0.008
IL-1β, pg/mL	167.85 ^a^	164.52 ^a^	110.53 ^b^	73.66 ^b^	16.77	0.001	0.000	0.001
IL-2, pg/mL	178.62	175.66	175.93	175.85	1.87	0.645	0.357	0.479
IL-4, pg/mL	4.54	5.13	5.75	4.29	0.80	0.586	0.892	0.417
IL-6, pg/mL	132.86 ^a^	112.63 ^ab^	104.23 ^b^	103.58 ^b^	4.15	0.005	0.000	<0.001
IL-10, pg/mL	4.33 ^c^	4.94 ^c^	9.88 ^a^	5.76 ^b^	0.41	<0.001	0.048	0.000
IFN-γ, pg/mL	42.08 ^a^	39.38 ^a^	35.43 ^b^	32.83 ^b^	1.28	0.000	<0.001	<0.001
TNF-α, pg/mL	35.22	33.67	35.91	31.70	5.72	0.971	0.805	0.949

^a–c^ Data with different letters in the same row indicate significant differences (*p* < 0.05). Abbreviations: NO = nitric oxide, iNOS = nitric oxide synthase, IL = interleukin, TNF-α = tumor necrosis factor-α, IFN-γ = interferon-γ.

**Table 6:** The effects of selenium yeast supplementation on selenium contents in milk and plasma.

Item	SY, mg/kg (DM Basis)				SEM	*p*-Value		
	0	0.15	0.3	0.5		Traetment	Linear	Quadratic
Plasma Se, ug/L	107.20 ^d^	157.20 ^c^	184.33 ^b^	217.58 ^a^	6.29	<0.001	<0.001	<0.001
Milk Se, ug/L	9.01 ^d^	24.07 ^c^	43.40 ^b^	58.75 ^a^	2.28	<0.001	<0.001	<0.001
(Milk/plasma Se), % ratio ^1^	7.79 ^c^	12.16 ^b^	26.80 ^a^	30.71 ^a^	2.82	<0.001	<0.001	<0.001

^a–d^ Data with different letters in the same row indicate significant differences (*p* < 0.05). ^1^ (Milk/plasma Se), % ratio = the ratio of milk-to-plasma Se, which is calculated by: milk Se content (ug/L)/plasma Se content (ug/L) × 100.

## Data Availability

The data presented in the current study are available from the corresponding author on reasonable request.
